# The association of motor imagery and kinesthetic illusion prolongs the effect of transcranial direct current stimulation on corticospinal tract excitability

**DOI:** 10.1186/s12984-016-0143-8

**Published:** 2016-04-15

**Authors:** Fuminari Kaneko, Eriko Shibata, Tatsuya Hayami, Keita Nagahata, Toshiyuki Aoyama

**Affiliations:** Laboratory of Sensory Motor Science and Sports Neuroscience, First Division of Physical Therapy, Sapporo Medical University, West 17- South 1, Chuo-ku Sapporo City, Japan; Development Research Group for Advanced Neuroscience-based Rehabilitation, Sapporo Medical University, West 17- South 1, Chuo-ku Sapporo City, Japan; Division of Health Science Education, School of General Education, Shinshu University, Asahi 3-1-1, Matsumoto City, Japan; Noboribetsu Hospital, Noboribetsuonsencho133, Noboribetsu City, Japan; Department of Physical Therapy, Ibaraki Prefectural University of Health Sciences, 4669-2 Ami, Ami-machi, Inashiki-gun Ibaraki, Japan

**Keywords:** Transcranial direct current stimulation, Motor imagery, Kinesthetic illusion, Visual stimulation, Transcranial magnetic stimulation, Corticospinal tract

## Abstract

**Background:**

A kinesthetic illusion induced by a visual stimulus (KI) can produce vivid kinesthetic perception. During KI, corticospinal tract excitability increases and results in the activation of cerebral networks. Transcranial direct current stimulation (tDCS) is emerging as an alternative potential therapeutic modality for a variety of neurological and psychiatric conditions, such that identifying factors that enhance the magnitude and duration of tDCS effects is currently a topic of great scientific interest. This study aimed to establish whether the combination of tDCS with KI and sensory-motor imagery (MI) induces larger and longer-lasting effects on the excitability of corticomotor pathways in healthy Japanese subjects.

**Methods:**

A total of 21 healthy male volunteers participated in this study. Four interventions were investigated in the first experiment: (1) anodal tDCS alone (tDCSa), (2) anodal tDCS with visually evoked kinesthetic illusion (tDCSa + KI), (3) anodal tDCS with motor imagery (tDCSa + MI), and (4) anodal tDCS with kinesthetic illusion and motor imagery (tDCSa + KIMI). In the second experiment, we added a sham tDCS intervention with kinesthetic illusion and motor imagery (sham + KIMI) as a control for the tDCSa + KIMI condition. Direct currents were applied to the right primary motor cortex. Corticospinal excitability was examined using transcranial magnetic stimulation of the area associated with the left first dorsal interosseous.

**Results:**

In the first experiment, corticomotor excitability was sustained for at least 30 min following tDCSa + KIMI (*p* < 0.01). The effect of tDCSa + KIMI on corticomotor excitability was greater and longer-lasting than that achieved in all other conditions. In the second experiment, significant effects were not achieved following sham + KIMI.

**Conclusions:**

Our results suggest that tDCSa + KIMI has a greater therapeutic potential than tDCS alone for inducing higher excitability of the corticospinal tract. The observed effects may be related to sustained potentiation of resultant cerebral activity during combined KI, MI, and tDCSa.

## Background

We previously reported that kinesthetic illusion (KI) [1–3] induced by visual stimuli (e.g., a video) can produce vivid kinesthetic perceptions in healthy subjects and patients with stroke at rest. Using transcranial magnetic stimulation, we found that corticospinal tract excitability increases concurrently with changes in the subjective feeling of kinesthetic perception [1, 2]. Furthermore, the activation of cerebral networks related to movement execution has been reported during the passive experience of KI [3]. Conversely, voluntary motor imagery has also been documented to increase the excitability of the corticospinal tract and activity in motor association areas [4–6]. Motor imagery (MI) describes the conscious and active psychological representation of movement. MI thus results in the activation of movement execution-related neural networks in healthy subjects [7–10]. In patients with stroke, increases in corticospinal tract excitability and activation in motor association areas in the involved cerebral hemisphere can improve the rehabilitation of sensory-motor function [11]. Therefore, a number of clinical studies have been conducted to assess different approaches for achieving this goal in stroke patients [12, 13]. Unfortunately, changes in the excitability of the corticospinal tract are typically short-lived or occur only during movement execution. Thus, there is a need for the development of interventions that can produce prolonged increases in corticospinal tract excitability in stroke patients.

Noninvasive transcranial direct current stimulation (tDCS) is a safe method for the selective modulation of local cerebral cortex excitability, and has recently received attention for its potential clinical utility [14]. A number of studies have reported that tDCS can affect brain plasticity and function. The magnitude and duration of the tDCS after-effect appear to depend on stimulation duration and current intensity; for example, in one study, 13 min of stimulation induced a 90-min after-effect [15]. In healthy subjects, tDCS acutely improves motor performance [16–19] and motor learning [16, 20–22]. Furthermore, a number of studies have reported that anodal tDCS, which involves the placement of an anode electrode over the motor area of the affected side and a cathode electrode above the contralateral orbit, acutely and chronically improves motor performance in patients with stroke [23–30]. Alternatively, cathodal tDCS, which uses an opposite configuration of anodal tDCS, has been reported to decrease corticospinal tract excitability in healthy subjects [31, 32]. Combinations of interventions (e.g., tDCS applied concurrently with another manipulation such as peripheral nerve stimulation) have also produced remarkable effects on motor performance in patients with stroke [33–35] and increased corticospinal tract excitability in healthy subjects [36].

We therefore hypothesized that the combination of MI and KI with tDCS would induce greater effects on corticospinal excitability than MI, KI, or tDCS alone. We also investigated whether the combination of MI and KI with tDCS would increase the magnitude and duration of corticospinal tract excitability. Anodal tDCS with KI and MI (tDCSa + KIMI) is a promising potential therapy for the clinical rehabilitation of stroke patients. Therefore, we examined the effect characteristics of combined tDCSa + KIMI therapy on corticospinal pathways in healthy Japanese subjects.

## Methods

### Participants

A total of 21 healthy male volunteers participated in the present study (average ± SD: age, 22.5 ± 1.0 years; height, 173.9 ± 5.7 cm; weight, 68.9 ± 7.8 kg). An all-male population resulted from the recruitment of subjects solely from our university. Two experiments were performed with an interval of 11 months between experiments. Twelve subjects participated in the first experiment and were asked to participate in the second experiment; however, only three of the twelve original subjects returned to participate in the second experiment due to the time interval between experiments in our study. The second experiment also included a total of twelve subjects. All subjects were right-handed according to the Oldfield handedness inventory [37]. Each subject provided informed consent for participation in the study, and all study protocols were approved by the Institutional Ethics Committee of Sapporo Medical University.

### Intervention

In the first experiment, each subject received four interventions in a randomized order to evaluate the effectiveness of combining tDCS with KI and/or MI. The interventions were as follows: (1) anodal tDCS alone (tDCSa), (2) anodal tDCS with kinesthetic illusion (tDCSa + KI), (3) anodal tDCS with motor imagery (tDCSa + MI), and (4) anodal tDCS with kinesthetic illusion and motor imagery (tDCSa + KIMI). Each intervention lasted 15 min, was conducted on a separate day per subject, and was repeated after a minimum interval of 1 week. In the tDCSa + KIMI condition, cerebral network activation was induced by tDCS together with visual stimulation and voluntarily executed MI: the subject was instructed to imagine self-movement similar to that in the movie being watched (visual stimulation). Visual stimulation was considered to induce strong KI if a subject moved their hands during the task. In the second experiment, we included an additional control group to confirm the effect of KIMI with sham stimulation (sham + KIMI).

**Fig. 1:**
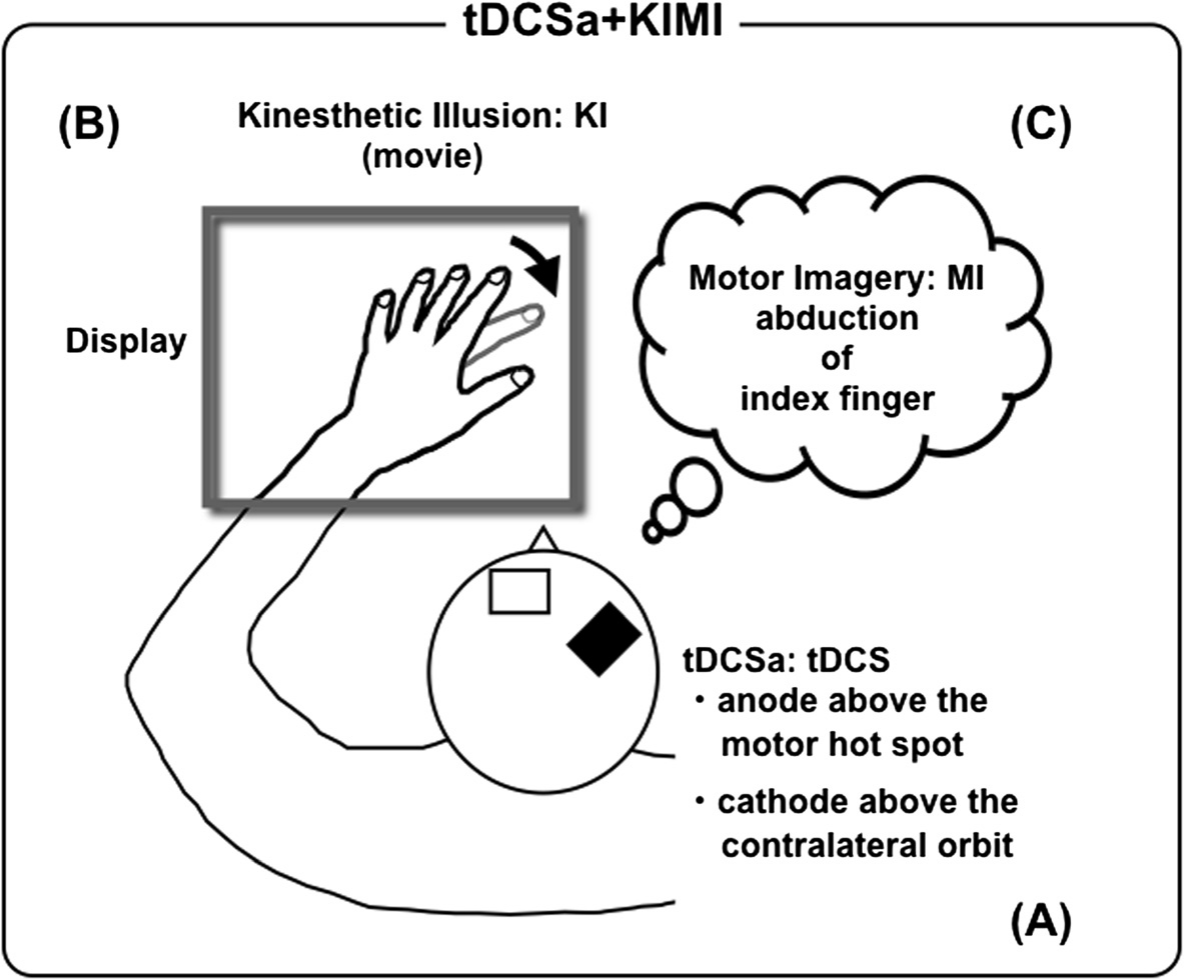
Schematic representation of multiple synchronized stimulations. Anodal tDCS was applied. An anode was placed above the motor hotspot of the left FDI and a cathode was placed above the contralateral orbit (**a**). A visual illusion was induced by having subjects view a movie of someone else’s index finger performing abduction/adduction (**b**). The subjects performed motor imagery of index finger abduction of their own index fingers (**c**)

Direct currents were applied through a pair of saline-soaked surface sponge electrodes (surface area, 35 cm^2^) and delivered using a battery-driven constant-current stimulator (DC-Stimulator, NeuroConn GmbH, Ilmenau, Germany) with a maximum output of 2 mA. For anodal tDCS, the center of each anode electrode was placed over the motor area for the left FDI and the cathode electrode was placed above the contralateral orbit (Fig. 1). Electrode positions were based on the results of a number of previous studies [31, 38–42]. In the stimulation period, DC current was gradually increased over a 7-s period until it reached 1 mA, and constant current stimulation was subsequently maintained for a total of 15 min. In the sham + KIMI condition, the DC current was gradually increased over a 7-s period until it reached 1 mA and subsequently decreased over a 7-s period to 0 mA. Therefore, sham + KIMI subjects received a 0 mA (sham) stimulation for a total of 15 min. The movie designed to produce the KI was filmed prior to the experiment. The performer, who did not participate in the TMS experiment, sat in a comfortable chair with his left hand on an experimental table in the same manner as subjects would in the TMS experiment. The physique and skin color of the performer were characteristic of an average Japanese male. A digital video camera (HDR-HC9, Sony Style (Japan) Inc., Tokyo, Japan) was carefully positioned at eye level beside the performer’s face, such that the camera angle would simulate the perspective of the performer’s eyes while watching his index finger move. Initially, active abduction of the index finger was performed (i.e., the index finger was spread away from the midline), followed by repeated adduction/abduction movements (i.e., the index finger was repeatedly spread open and squeezed closed). The duration of an entire movement cycle (e.g., neutral to full abduction to full adduction) lasted 6 s, and the movie was played on a loop. In conditions including visual stimulation and tDCS, the movie was initiated at the beginning of tDCS and terminated at the end of tDCS. The KI was created as previously described [1, 3]. Briefly, a liquid crystal display (FlexScan S1961, Nanao Inc., Ishikawa, Japan) on which the movie was played (QuickTime ver. 7.6, Apple Computer, Inc., CA, USA) was affixed in an appropriate position on the subject’s forearm and the movie window was resized so that the performer’s hand size closely matched the subject’s hand size and location. The visual analog scale (VAS) was used to obtain a measure of introspective perception of movement while watching the movie in the illusion condition, as previously described [1]. Before and after the KI session, subjects were interviewed about the strength of the kinesthetic illusion; subjects provided answers on the VAS regarding the strength of the feeling of actual movement, with “0 mm” indicating that subjects had no illusory sensation at all and “100 mm” indicating that they felt exactly as if their own finger was moving. Before the KI session, we instructed KI training and confirmed that all subjects reported a VAS score greater than 50 mm.

**Fig. 2:**
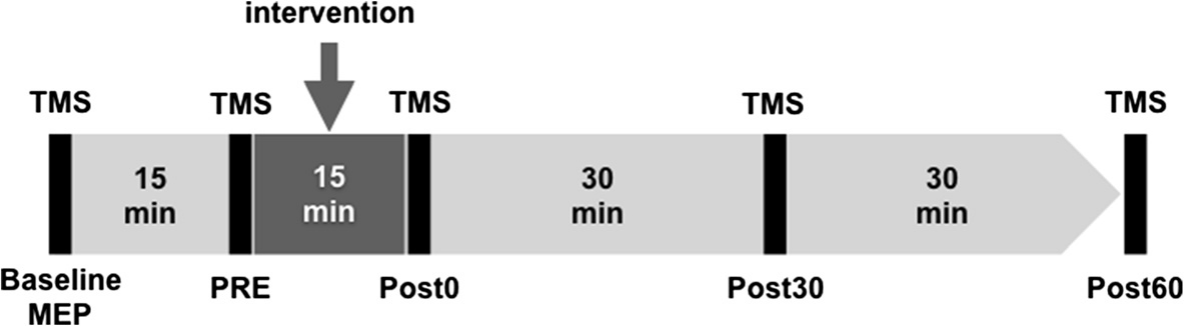
Experimental timeline. Interventions were conducted for 15 min each. Cortical excitability was examined using transcranial magnetic stimulation (TMS) prior to the intervention to establish control conditions (Baseline MEP = pre-intervention, PRE = 15 min after baseline MEP) and at timed intervals after the intervention (Post0 = 0 min post-intervention, Post30 = 30 min, and Post60 = 60 min). We calculated the ratio of MEP amplitudes at each post-intervention time-point relative to baseline MEPs [(MEP amplitude at each stage – baseline MEP amplitude)/baseline MEP amplitude × 100]

MI [43] was performed as previously described. Briefly, subjects were directed to imagine index finger abduction. The subject was asked to remember the perception and scene associated with muscle contraction for index finger abduction, as explained in our previous study [44]. MI was repeated similarly to the KI condition: index finger abduction for 3 s with a 3-s pause was repeated for 15 min.

### Procedures and TMS examination

Each subject sat with his left hand fixed to an experimental table. Surface electromyograms (EMGs) were recorded from the first dorsal interosseous (FDI) of the left hand with 8-mm-diameter Ag/AgCl-plated surface electrodes using a belly-tendon montage. EMG signals were amplified (Neuropack MEB2200, Nihonko-den Co. Ltd., Tokyo, Japan) to an appropriate level and band-pass filtered at 5–1000 Hz. All signals were sampled at 20 kHz from 500 ms before to 500 ms after the delivery of the stimulus using an A/D converter (Power 1401 with Signal 2.14 software, Cambridge Electronic Design, Cambridge, UK) and stored on a computer. EMG signals were observed on a 17-in. computer display in real-time to identify any tiny muscular contractions (EMG signals with apparent amplitudes exceeding 20 μV) and a trial was rejected if a small level of muscle activation was observed during the testing. Furthermore, each trial was examined off-line, and trials containing EMG signals with amplitudes that exceeded 20 μV were excluded from the data analysis. TMS was delivered over the right primary motor cortex using a single pulse monophasic stimulator with a figure-eight-shaped magnetic coil (9 cm diameter for each loop; Magstim 200^2^, The Magstim Company Limited, Whitland, UK). Motor-evoked potentials (MEPs) were recorded after each intervention. TMS procedures were performed according to standard guidelines [45, 46]. A TMS mapping session was performed to identify and functionally define the left FDI and abductor digiti minimi (ADM) hotspots (i.e., the points where stimulation generated the largest magnitude MEPs from the FDI and ADM). The test TMS pulses were set at an intensity that produced a MEP of about 1 mV in the baseline MEP test. The baseline MEP test and other MEP tests during each examination stage were performed by delivering 10 pulses every 6–8 s at each time-point. The MEP test before the intervention (PRE) was performed 15 min after the baseline MEP test to confirm that MEPs were not spontaneously altered over time. After the intervention, cortical excitability was assessed every 30 min at specific time intervals (Post0 = 0 min post-intervention, Post30 = 30 min post-intervention, and Post60 = 60 min post-intervention; Fig. 2).

### Data analysis

MEP magnitudes were determined by averaging peak-to-peak amplitudes. The ratio of the MEP amplitude recorded from the FDI (MEP ratio) at each post-intervention time-point relative to the baseline MEP was calculated [(MEP amplitude at each stage – baseline MEP amplitude)/baseline MEP amplitude × 100]. Statistical analyses were performed on the calculated test-to-baseline MEP ratios. In the first experiment, a two-way repeated measures ANOVA was used to test the main effects of “experimental time-point” (PRE, Post0, Post30 and Post60) and “intervention” (tDCSa, tDCSa + KI, tDCSa + MI, and tDCSa + KIMI). To further investigate the effect of experimental time-point, we used Dunnett’s test, which is specifically designed for the examination of data with respect to a single set of reference data (PRE). Tukey’s honest significant difference test was used to further evaluate the effect of intervention. In the second experiment, one-way repeated measures ANOVA was used to test the effect of “time-point”. Dunnett’s test was used to further examine MEP amplitudes with respect to experimental time-point. The significance level was set at *p* < 0.05.

## Results

The average response on the VAS after KI was 71.4 ± 13.4 mm, indicating that subjects felt as though their own finger was actually moving while viewing the KI movie.

**Fig. 3:**
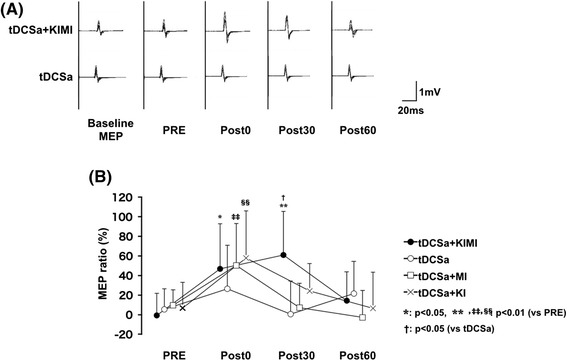
The increase in MEP amplitudes under combination with tDCS. Superimposed raw EMGs of MEPs recorded from a single subject’s FDI in the tDCSa + KIMI and tDCSa conditions are shown (**a**). Results of the average MEP ratio from the FDI induced in each of the 4 conditions are shown for each time-point (**b**). Error bars indicate the standard deviation. *: *p* < 0.05 vs. PRE, **, ‡‡, §§: *p* < 0.01 vs. PRE (Dunnett’s post-hoc test). †: *p* < 0.05 vs. tDCSa (Tukey’s post-hoc test)

Increases in MEP amplitudes were recorded from the FDI at Post0 and Post30 in the tDCSa + KIMI condition (Fig. 3a). In contrast, MEP amplitudes after tDCSa alone were not significantly affected (Fig. 3a). Fig. 3b shows the average (± SD) ratios of MEPs induced by each intervention in the first experiment. A two-way repeated measures ANOVA indicated significant main effects of experimental time-point on the ratio of MEP amplitudes from the FDI [F (1.474, 16.219) = 10.661, *p* = 0.002]; on the other hand, there were no significant main effects for intervention [F (3, 33) = 2.068, *p* = 0.123]. The interaction between experimental time-point and intervention was also significant [F (3.658, 40.234) = 3.465, *p* = 0.018]. Dunnett’s post-hoc test revealed that, in the tDCSa + KIMI group, MEP ratios were significantly larger in the Post0 and Post30 time-points relative to the PRE time-point. In the tDCSa condition, the MEP ratio was not significantly affected post-session. In the tDCSa + MI and tDCSa + KI conditions, MEP ratios at Post0 were significantly larger than those at PRE. Tukey’s honest significant difference test revealed that the MEP ratio in the tDCSa + KIMI condition was larger than that in the tDCSa condition at Post30 (*p* = 0.022). No other significant differences were observed.

**Fig. 4:**
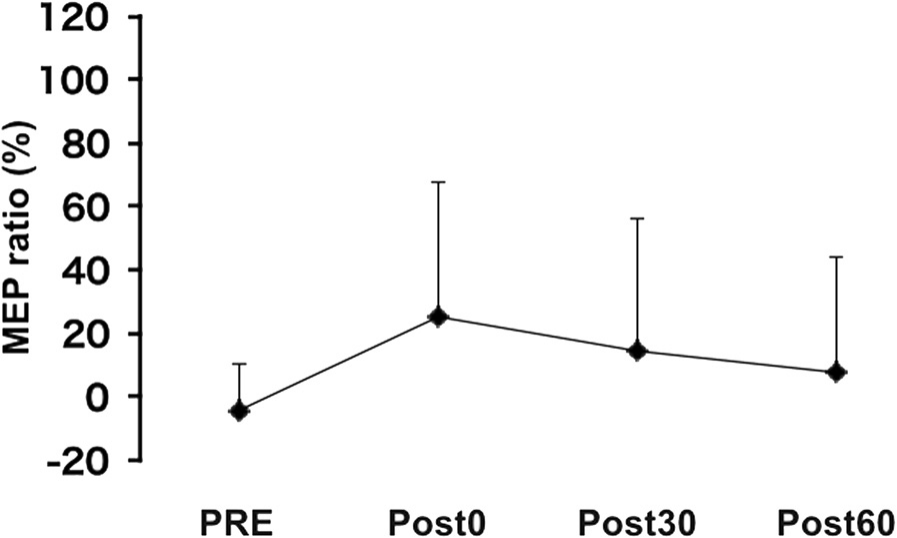
The MEP ratio in the sham + KIMI Results of the average MEP ratio of FDI induced in the sham + KIMI conditions at each time-point. Error bars indicate the standard deviation

In the second experiment, one-way repeated measures ANOVA indicated no significant main effects of experimental time-point on the ratio of MEP amplitudes from the FDI [F (3, 33) = 1.575, *p* = 0.214; Fig. 4].

## Discussion

The results of this study can be summarized as follows: (i) tDCSa + KIMI induced changes in corticospinal tract excitability associated with the FDI that were longer-lasting than changes in other conditions, (ii) corticospinal tract excitability was sustained for at least 30 min after tDCSa + KIMI, and (iii) MI and KI interventions without tDCS (sham + KIMI) did not induce significant MEP facilitation effects.

The duration of enhanced corticospinal tract excitability induced in the present study with tDCSa + KIMI was longer than in other conditions. Nonetheless, the duration we observed was shorter than that reported in previous studies using tDCS [31, 38–42]. Previous studies have reported increases ranging between +20 % and +50–60 % relative to baseline with durations of 30–60 min [31, 38–42]. In the present study, we observed a relative change in corticospinal tract excitability of about +60 %, but a shorter duration of the effect. What accounts for this discrepancy is currently unknown and is beyond the scope of the current study. Regardless, we found that the combined tDCSa + KIMI condition was more effective at augmenting corticospinal tract excitability than non-combinatorial conditions.

The present study tested whether the application of tDCSa + KIMI could produce stronger excitability effects than tDCS alone. One research group previously reported that tDCS-mediated effects are more difficult to achieve in Japanese cohorts than in a Western populations [47]. In this report, we observed significant effects in a Japanese cohort as early as 1 and 15 min after tDCS; however, while the largest change in relative MEP amplitudes was almost twice that of the baseline, this effect was not significant at 0, 3, 5, 10, or 20 min after tDCS alone. That is, in the present study, the effect of independent anodal tDCS was not significant. These findings are in agreement with those of Furubayashi et al. (2008). Additionally, very recently, a large range in responses to tDCS has been reported [48], which corroborates some aspects of our results. Additional studies evaluating the use of tDCS with a 1 mA intensity (particularly in Japanese subjects) are required.

The enhancement of corticospinal tract excitability after independent tDCSa was not statistically significant; however, enhanced MEP amplitudes after tDCSa + KIMI may indicate that tDCSa causes longer effect of KIMI on the corticospinal excitability enhancement. An absence of effect in the sham + KIMI condition suggested that our observed effect in the tDCSa + KIMI condition required tDCS anodal stimulation to produce persistent changes in corticospinal tract excitability. Differences between the tDCSa + KIMI and sham + KIMI conditions suggested that a combination of tDCS with MI and KI are decisive factor for the observed effect.

Although we have not explored mechanisms underlying the enhancement of corticospinal tract excitability, we propose that Hebb’s rule [49] may at least partly explain the current results. In the last decade, associative long-term potentiation induced by paired associative stimulation (PAS), which combines repetitive peripheral nerve stimulation with TMS, has been investigated in human subjects [50–52]. Based on previous studies in which activation of the cerebral cortex and the corticospinal tract were reported during MI and KI, presynaptic input may have superimposed upon the effects of tDCS in the present study [1–5, 9, 44, 53–58]. Indeed, a number of brain imaging studies have reported activation of similar motor areas during MI and actual movement [8, 10, 7, 59], as well as the activation of associated areas during KI induced by tendon vibration [53, 56, 60, 61]. KI induced by visual stimulation has also been reported to alter corticospinal tract excitability [1]. We recently observed that areas activated during visually-evoked KI include higher motor association areas, such as the premotor and supplementary motor cortices [3]. Furthermore, bihemispheric cortical stimulation results in the synergistic modulation of cerebral network blood flow and cerebral network excitability [62]. We therefore propose that, consistent with previous results [62], the combination of MI and KI with tDCS has a synergistic effect on cerebral network activation.

The effects of tDCS have been hypothesized to involve sodium and calcium channels in an *N*-methyl-D-aspartate (NMDA) receptor-dependent manner [63]. Moreover, a recent study suggested that tDCS reorganizes the intrinsic architecture of the primary motor cortex [64]; NMDA receptor-dependent alterations of synaptic strength may be involved in this mechanism. Consequently, it may be possible that a longer-lasting facilitatory effect produced in the tDCSa + KIMI group resulted from sodium and calcium channel conductance changes, and/or NMDA receptor modulation. Since the intervention effect associated with MI and/or KI was markedly shorter in the absence of tDCS, we speculate that the role of cerebral network activity during MI and/or KI was to consolidate the effects of tDCS.

There are several ways to non-invasively induce corticomotor plasticity in the clinical setting [47, 50, 65–75]. In terms of clinical utility, tDCS has several advantages relative to other brain stimulation techniques such as repetitive TMS. These advantages include decreased patient distress, ease of administration without precise mapping or the establishment of motor thresholds, and equipment portability. Additionally, because interventions that persistently modulate cortical excitability are important for stroke rehabilitation, our demonstration that tDCSa + KIMI might induce long-lasting changes in excitability improves the therapeutic potential of this approach [25, 26, 76]. Since motor learning processes are accompanied by cortical excitability shifts and by changes of synaptic efficacy [77], it is very likely that larger effects on corticospinal tract excitability demonstrated in this study could significantly enhance motor learning and performance in rehabilitation.

A major limitation of this study is the low number of participants, which may have compromised our ability to detect statistically significant differences. However, we believe that our results show an intriguing potential for the combination of KI, MI, and tDCS in a therapeutic modality. The present results warrant future study with a larger patient cohort for validation.

## Conclusions

The present study suggests that tDCSa + KIMI has a greater therapeutic potential than tDCS alone for inducing primary motor cortex excitability. Excitability related to tDCSa + KIMI may involve sustained potentiation resultant from the convergence of cerebral network activity during KI and MI with tDCSa effects on corticospinal tract excitability. This approach constitutes an interesting possibility for the future of neuroscience-based rehabilitation, and has potential applications in a variety of patient populations including those of stroke and chronic pain.

## Abbreviations

ADM: abductor digiti minimi
EMG: electromyogram
FDI: first dorsal interossei
MEP: motor-evoked potentials
NMDA: *N*-methyl-D-aspartate
RMT: resting motor threshold
sham + KIMI: combined stimulation of sham tDCS with MI and KI
tDCS: transcranial direct current stimulation
tDCSa: anodal tDCS
tDCSa + KI: anodal tDCS with kinesthetic illusion
tDCSa + KIMI: anodal tDCS with kinesthetic illusion and motor imagery
tDCSa + MI: anodal tDCS with motor imagery
